# The Story of the Insane from Year to Year

**Published:** 1900-12-01

**Authors:** 


### THE STORY OF THE INSANE FROM YEAR TO YEAR.

(THIRTEENTH SERIES.)

### THE OXFORD COUNTY ASYLUM AT LITTLEMORE

ON January 1st, 1899, this asylum contained 533 patients and by the end of the year the number had risen to 552. There were 109 admissions, 24 re-admissions, 34 recoveries, 11 discharged relieved, and 59 deaths. The percentage of recoveries on the year's admissions is only 20, and the percentage of deaths on the average number resident is 11; the first being very low and the latter rather high. These figures are taken from Table V., on page 43; but it would seem from Dr. Sankey's Report on page 36 that the recoveries reached 29 per cent. of the admissions. Moral causes account for the insanity of the cases admitted in 8, intemperance in 12, and hereditary influence in 20. Cerebral and spinal diseases account for 20 of the deaths; phthisis for 3; pneumonia for 3; enteritis for 1 and typhoid fever for 1. There is again a conflict of opinion on several points between the Commissioners in Lunacy and the Committee of Visitors. The former say there is not really accommodation for 534 patients and the latter say there is. The most important thing is that the Commissioners say, "We had complaints of rough usage at the hands of nurses and attendants, and we think there may be some grounds for these complaints." The Committee say, "the superintendent assures the sub-committee that the only cases alleged of rough usage which the Commissioners mentioned to him, or of which he himself was aware, were one which occurred in April, 1895, to S. W., into which the Commissioners, in conjunction with himself, made full inquiry, and another to A. W., who received a slight bruise on the hand in a scuffle with her attendants, and which she acknowledged to have been caused by her own fault." We make no comment on these charges and answers. The visitors say that "the conduct and efficiency of the several officers, attendants, and servants of the Asylum during the past year has been wholly satisfactory." The weekly charge to the Unions was 7s. 7d. per head.

### THE GLASGOW ROYAL ASYLUM AT GARTNAVEL.

ON January 1st, 1899, there were 424 patients in residence and by December 31st the number had risen to 427. There were 104 first admissions, 31 re-admissions, 54 recoveries, 55 discharged relieved, and 23 deaths. Calculated in the usual way in asylums, the recoveries stand at 40 per cent. and the deaths at 5·5 per cent. The first of these is high and the second low; but it would hardly be fair to compare county or district asylums with the Royal or private asylums, because there is considerable difference in the classes of patients admitted. Of the deaths 7 were caused by cerebral or spinal diseases, 3 by pneumonia, and only 1 by phthisis. Of the year's admissions 23 owed their insanity to such moral causes as domestic or business troubles, religious excitement, &c., 25 to intemperance in drink, 24 to previous attacks, and 2 to hereditary influence. The latter seems absurdly small when dealing with 135 admissions. Dr. Yellowlees says that "chronic insanity, if not associated with obvious and progressive brain diseases, does not tend to shorten life in asylum patients." Judging from the great ages reached by many asylum inmates one is almost tempted to believe that the absence of worry and the quiet seclusion enjoyed by the chronic lunatic tend to lengthen life in very many cases. On the great nursing question we extract the following sentences from Dr. Yellowlees' Report: "Mental nursing is far higher and more difficult work than the nursing of mere bodily illness. It requires the highest qualities of head and heart; and it is a matter for regret that ladies who now occupy themselves so frequently in ordinary sick-nursing hold so aloof from this work which needs them so much." In England, at least, a beginning has been made, and when once asylum nursing becomes really fashionable. Dr. Yellowlees will be besieged in the same reckless way that hospital authorities now are. The charges for private patients range from £40 up to £400; and the directors state that "the charitable element in the administration of the institution receives the constant attention of the directors. It is exercised not only by admitting special cases at unremunerative rates; but by maintaining in their former grade patients who have long been unable to pay for the same." This is all very well as far as it goes; but we should like to hear something about the numbers received under these conditions, and the amount paid by each. A table showing these facts in the next annual report is much to be desired.

